# A Rare Case of *t*(1; 19) Translocation in Acute Myeloid Leukemia: Evaluation of a High-Risk Patients

**DOI:** 10.1155/crh/4655315

**Published:** 2025-11-06

**Authors:** Songül Beskisiz Dönen, Abdullah Karakuş, Mehmet Orhan Ayyıldız

**Affiliations:** Hematology Department, Faculty of Medicine, Dicle University, Diyarbakır, Türkiye

**Keywords:** acute myeloid leukemia, high-risk AML, *t*(1; 19)

## Abstract

Acute Myeloid Leukemia (AML) is a heterogeneous malignancy arising from the malignant transformation of hematopoietic stem cells, characterized by the accumulation of blasts in the myeloid lineage. While common cytogenetic alterations in AML play a critical role in prognostic classification, rare chromosomal translocations may have distinct impacts on disease biology and treatment response. In this report, we present a high-risk AML case harboring the *t*(1; 19) translocation. By highlighting the diagnostic and therapeutic challenges posed by this uncommon genetic aberration, this case aims to contribute to clinical practice in the field of hematology.

## 1. Introduction

Acute myeloid leukemia (AML) is a heterogeneous malignant disease characterized by clonal proliferation of hematopoietic stem cells and accumulation of immature myeloid cells in the bone marrow, peripheral blood, and various tissues. The clinical course of AML varies depending on its genetic subtypes. It is the most common form of acute leukemia in adults, and its incidence increases with age [1, 2].

Although the diagnosis is generally based on elevated blast counts in the bone marrow and characteristic immunophenotypic findings, certain AML subtypes may be diagnosed even with blast percentages below 20%. Therefore, in addition to morphological evaluation, genetic and molecular analyses have become integral components of the diagnostic process [3]. Common chromosomal rearrangements (such as *t*(8; 21), inv(16), and *t*(15; 17)) and mutations (including NPM1, FLT3, and CEBPA) play critical roles in prognostic assessment [4, 5]. The ELN and WHO classifications use these genetic data to define risk groups and guide treatment strategies [6, 7].

The *t*(1; 19)(q23; p13) translocation is a well-recognized genetic abnormality primarily associated with B-ALL, resulting in a fusion between the E2A (TCF3) and PBX1 genes. This translocation disrupts transcriptional regulation in leukemic cells, promoting increased proliferation. Its occurrence in AML is rare and thus clinically noteworthy [8–11].

Detection of the *t*(1; 19) translocation in AML typically places the patient in the high-risk category and necessitates a more aggressive treatment approach. Therefore, its role in differential diagnosis, clinical impact, and treatment implications warrants special consideration [12]. In this case report, we present the diagnostic process, morphological and molecular features, and treatment approach of a rare AML case harboring the *t*(1; 19)(q23; p13) translocation.

This case report has been prepared in accordance with the CARE guidelines.

## 2. Case Presentation

A 35-year-old female patient presented with complaints of fatigue and generalized body pain. Complete blood count revealed leukocytosis (16,980/μL; reference range: 4000–10,000/μL), neutrophilia (8880; 1800–7700/μL), severe anemia (hemoglobin: 6.1; 12–16 g/dL), low hematocrit (19.2%; 36%–48%), thrombocytopenia (22,000; 150,000–450,000/μL), and monocytosis (3630; 200–1000/μL). Peripheral blood smear demonstrated numerous circulating blasts. Abdominal ultrasonography showed hepatomegaly.

**Figure 1:**
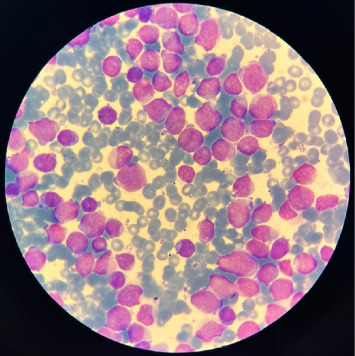
Bone marrow aspirate demonstrating blasts consistent with AML.

Bone marrow evaluation revealed extensive infiltration with blast cells. Immunohistochemical analysis was positive for CD34, CD33, myeloperoxidase (MPO), and CD117. The morphology, elevated blast count, and immunophenotype were consistent with a diagnosis of myeloproliferative neoplasm (Figure 1).

Molecular studies revealed negative results for FLT3-ITD, IDH1, IDH2, C-KIT, and inv(16), while NPM1 mutation was detected as positive. Cytogenetic analysis showed no evidence of *t*(8; 21) or *t*(15; 17); however, the rare *t*(1; 19) translocation was identified. Based on these findings, a diagnosis of AML was established, and the patient was categorized in the high-risk group.

Induction chemotherapy with the standard 7 + 3 regimen (cytarabine 100 mg/m^2^/day for 7 days plus an anthracycline 12 mg/m^2^/day for 3 days) was initiated. Following identification of a fully HLA-matched sibling donor, the patient underwent consolidation therapy with high-dose cytarabine (HDAC, 3 g/m^2^) and was subsequently referred to a specialized center for allogeneic hematopoietic stem cell transplantation (allo-HSCT). Unfortunately, disease relapse occurred within the first 6 months post-transplant, and a second transplant was planned.

## 3. Discussion

Acute myeloid leukemia (AML) is a biologically heterogeneous malignancy classified into distinct subgroups based on genetic and molecular alterations. The clinical course and treatment responses are largely influenced by these genetic abnormalities [1–6]. The *t*(1; 19)(q23; p13) translocation, which typically results in an E2A-PBX1 fusion, is a well-characterized cytogenetic abnormality predominantly associated with acute lymphoblastic leukemia (ALL). In contrast, its occurrence in AML is exceptionally rare and has been documented in only a limited number of case reports [8–11].

To date, most published data on *t*(1; 19)-positive AML stem from isolated case reports, leaving the clinical implications, treatment responses, and prognostic significance of this translocation largely undefined. However, the available literature suggests that patients harboring this aberration often exhibit aggressive disease behavior, are classified within high-risk prognostic categories, and experience early relapse [8].

In our case, the *t*(1; 19) translocation was accompanied by an NPM1 mutation. While NPM1 mutations are generally associated with favorable outcomes in AML, the concomitant presence of *t*(1; 19) may have mitigated this beneficial effect and contributed to a more adverse clinical course.

Given the presence of *t*(1; 19) at diagnosis, our patient was stratified into the high-risk group and received standard 7 + 3 induction chemotherapy (7 days of cytarabine and 3 days of an anthracycline). A complete remission was achieved post-induction, and high-dose cytarabine (HDAC; 1500 mg/m^2^/day for 3 days) was administered as consolidation therapy. Due to the availability of a fully HLA-matched sibling donor, the patient was referred to a tertiary center for allo-HSCT. Unfortunately, the disease relapsed within 6 months post-transplant, and a second transplantation was planned. This clinical course further supports the notion that the *t*(1; 19) translocation may serve as an adverse prognostic marker in AML.

This case highlights the clinical relevance of the rare *t*(1; 19) translocation in AML and suggests that current therapeutic approaches may be inadequate for this subgroup. The scarcity of reported cases underscores the need for multicenter studies with larger patient cohorts and integrated genetic profiling to better elucidate the biological behavior and optimal management strategies for *t*(1; 19)-positive AML.

## 4. Conclusion

This case illustrates the impact of the rare *t*(1; 19) translocation on the diagnostic and therapeutic trajectory in AML. *t*(1; 19) positivity represents a high-risk genetic alteration requiring more intensive treatment approaches. Intensive chemotherapy and planned allogeneic stem cell transplantation play a crucial role in achieving long-term remission in such cases.

In AML patients, comprehensive molecular testing that includes rare translocations typically associated with ALL is essential for accurate diagnosis and appropriate therapeutic planning. Early identification of such genetic alterations enables timely decisions that can significantly influence clinical outcomes.

With this case, we aim to raise awareness regarding the clinical management of rare cytogenetic abnormalities in AML and to contribute to the expanding literature in this domain.

## References

[1] Grimwade D., Karp J. E. (2007). Impact of Cytogenetics on Clinical Outcome in AML. *Acute Myelogenous Leukemia*.

[2] Mrózek K., Heerema N. A., Bloomfield C. D. (2004). Cytogenetics in Acute Leukemia. *Blood Reviews*.

[3] Grimwade D., Hills R. K. (2009). Independent Prognostic Factors for AML Outcome. *Hematology*.

[4] Grimwade D., Hills R. K., Moorman A. V. (2010). Refinement of Cytogenetic Classification in Acute Myeloid Leukemia: Determination of Prognostic Significance of Rare Recurring Chromosomal Abnormalities Among 5876 Younger Adult Patients Treated in the United Kingdom Medical Research Council Trials. *Blood*.

[5] Tang J. L., Hou H. A., Chen C. Y. (2009). AML1/RUNX1 Mutations in 470 Adult Patients with De Novo Acute Myeloid Leukemia: Prognostic Implication and Interaction with Other Gene Alterations. *Blood*.

[6] Gaidzik V. I., Bullinger L., Schlenk R. F. (2011). RUNX1 Mutations in Acute Myeloid Leukemia: Results from a Comprehensive Genetic and Clinical Analysis from the AML Study Group. *Journal of Clinical Oncology*.

[7] Arber D. A., Orazi A., Hasserjian R. (2016). The 2016 Revision to the World Health Organization Classification of Myeloid Neoplasms and Acute Leukemia. *Blood*.

[8] Troussard X., Rimokh R., Valensi F. (1995). Heterogeneity of *t*(1;19)(q23;p13) Acute Leukaemias. French Haematological Cytology Group. *British Journal of Haematology*.

[9] Pui C. H., Campana D., Pei D. (2009). Treating Childhood Acute Lymphoblastic Leukemia Without Cranial Irradiation. *New England Journal of Medicine*.

[10] Hunger S. P., Mullighan C. G. (2015). Acute Lymphoblastic Leukemia in Children. *New England Journal of Medicine*.

[11] Inaba H., Greaves M., Mullighan C. G. (2013). Acute Lymphoblastic Leukaemia. *The Lancet*.

[12] Papaemmanuil E., Gerstung M., Bullinger L. (2016). Genomic Classification and Prognosis in Acute Myeloid Leukemia. *New England Journal of Medicine*.

